# Supplementary material for: Volatile profiling distinguishes Streptococcus pyogenes from other respiratory streptococcal species
Source: mSphere. 2023 Oct 4;8(5):e00194-23. doi: 10.1128/msphere.00194-23 (PMC10597408; doi:10.1128/msphere.00194-23)
Supplement: Figure S1 — Normalized average abundance of the 27 discriminatory metabolites. [file msphere.00194-23-s0001.docx]

**Supplemental Material**

**Volatile profiling distinguishes *Streptococcus pyogenes* from other respiratory streptococcal species**

Amalia Z. Berna^1,2^, Joseph A. Merriman^3,4^, Leah Mellett^3^, Danealle K. Parchment^3,5^, Michael G. Caparon^3^, Audrey R. Odom John^1,2,6 *^

^1^Department of Pediatrics, Washington University School of Medicine, St. Louis, MO 63110, USA.

^2^Department of Pediatrics, Children’s Hospital of Philadelphia, Philadelphia, PA 19104, USA.

^3^Department of Molecular Microbiology, Washington University School of Medicine, St. Louis, MO 63110, USA.

^4^Microbiome Therapies, Stanford University, Palo Alto, CA 94304, USA

^5^Vaccine Research Center, National Institute of Allergy and Infectious Diseases, Bethesda, MD 20892, USA

^6^Perelman School of Medicine, University of Pennsylvania, Philadelphia, PA 19104, USA

Correspondence to: johna3@chop.edu

**Supplementary Figure 1**: Normalized average abundance of the 27 discriminatory metabolites identified after Kruskal-Wallis test across the 3 pathogen groups assessed in this study. Volatiles are groups in low, medium and high levels for easy interpretation. VOC numbers correspond to compounds reported in Table 1. *S. pyogenes* (**blue**), *S. intermedius* (**green**) *S. pneomonia* (**coral**).
